# Carpal Tunnel Syndrome: Neuropathic Pain Associated or Not with a Nociplastic Condition

**DOI:** 10.3390/biomedicines11061744

**Published:** 2023-06-17

**Authors:** César Fernández-de-las-Peñas, Stella Fuensalida-Novo, Jo Nijs, Annalie Basson, Gustavo Plaza-Manzano, Juan A. Valera-Calero, Lars Arendt-Nielsen, Ana I. de-la-Llave-Rincón

**Affiliations:** 1 Department of Physical Therapy, Occupational Therapy, Rehabilitation and Physical Medicine, Universidad Rey Juan Carlos, 28922 Alcorcón, Spain; stella.fuensalida@urjc.es (S.F.-N.); anaisabel.delallave@urjc.es (A.I.d.-l.-L.-R.); 2 Center for Neuroplasticity and Pain (CNAP), SMI, Department of Health Science and Technology, Faculty of Medicine, Aalborg University, DK-9220 Aalborg, Denmark; lan@hst.aau.dk; 3 Pain in Motion Research Group (PAIN), Department of Physiotherapy, Human Physiology and Anatomy, Faculty of Physical Education & Physiotherapy, Vrije Universiteit Brussel, 1050 Brussels, Belgium; jo.nijs@vub.be; 4 Chronic Pain Rehabilitation, Department of Physical Medicine and Physiotherapy, University Hospital Brussels, 1090 Brussels, Belgium; 5 Unit of Physiotherapy, Department of Health and Rehabilitation, Institute of Neuroscience and Physiology, Sahlgrenska Academy, University of Gothenburg, 405 30 Gothenburg, Sweden; 6 Department of Physiotherapy, University of the Witwatersrand, Office 23, Khanya Block-West, 7 York Road, Parktown 2193, South Africa; bassonannalie@gmail.com; 7 Department of Radiology, Rehabilitation and Physiotherapy, Complutense University of Madrid, 28040 Madrid, Spain; gusplaza@ucm.es (G.P.-M.); juavaler@ucm.es (J.A.V.-C.); 8 Grupo InPhysio, Instituto de Investigación Sanitaria del Hospital Clínico San Carlos (IdISSC), 28040 Madrid, Spain; 9 Department of Gastroenterology & Hepatology, Mech-Sense, Clinical Institute, Aalborg University Hospital, DK-9000 Aalborg, Denmark; 10 Steno Diabetes Center North Denmark, Clinical Institute, Aalborg University Hospital, DK-9000 Aalborg, Denmark

**Keywords:** carpal tunnel syndrome, median nerve, nociceptive, neuropathic, nociplastic pain, precision medicine, peripheral drive, central sensitization

## Abstract

Carpal tunnel syndrome (CTS) has been traditionally classified as primarily a neuropathic condition with or without pain. Precision medicine refers to an evidence-based method of grouping patients based on their susceptibility to biology, prognosis of a particular disease, or in their response to a specific treatment, and tailoring specific treatments accordingly. In 2021, the International Association for the Study of Pain (IASP) proposed a grading system for classifying patients into nociceptive, neuropathic, or nociplastic phenotypes. This position paper presents data supporting the possibility of subgrouping individuals with specific CTS related-pain into nociceptive, neuropathic, nociplastic or mixed-type phenotypes. Carpal tunnel syndrome is a neuropathic condition but can also be comorbid with a nociplastic pain condition. The presence of extra-median symptoms and the development of facilitated pain processing seem to be signs suggesting that specific CTS cases can be classified as the nociplastic pain phenotype. The clinical responses of therapeutic approaches for the management of CTS are inconclusive. Accordingly, the ability to identify the predominant pain phenotype in patients with CTS could likely be problematic for producing efficient treatment outcomes. In fact, the presence of a nociplastic or mixed-type pain phenotype would explain the lack of clinical effect of treatment interventions targeting the carpal tunnel area selectively. We propose a clinical decision tree by using the 2021 IASP classification criteria for identifying the predominant pain phenotype in people with CTS-related pain, albeit CTS being a priori a neuropathic pain condition. The identification of a nociplastic-associated condition requires a more nuanced multimodal treatment approach to achieve better treatment outcomes.

## 1. Introduction

Carpal tunnel syndrome (CTS) is the most prevalent compression disorder of the upper extremity. Although epidemiological data depend on the diagnostic criteria used [1], CTS has an incidence rate of 1.8/1000 [2] and an estimated lifetime prevalence of 3.1% [3]. This pain condition is more prevalent in women and mainly affects middle-aged workers; it is associated with substantial health care costs and loss of productivity [4].

Carpal tunnel syndrome is traditionally diagnosed by electrophysical examination of the median nerve conduction velocity [5]; however, evidence supports that CTS can be a complex syndrome associated with central neuronal excitability changes and altered pain modulation [6]. Previous studies have discussed the role of peripheral and central mechanisms in individuals with CTS [7,8]; nevertheless, it is relevant to adapt the nomenclature of neuropathic pain conditions, such as CTS, for current development in the field. The current paper includes these developments and thereby updates our previous review on this topic [7,8].

In 2016, three pain phenotypes, i.e., nociceptive, neuropathic and nociplastic pain, as well as a fourth one, i.e., a mixed type, were proposed [9]. The new term that was introduced at this point is nociplastic pain. Nociplastic pain is defined as “pain that arises from altered nociception despite no clear evidence of actual or threatened tissue damage causing the activation of peripheral nociceptors or evidence for disease or lesion of the somatosensory system causing the pain” [9]. Though this definition has become well established in the literature, it has also raised several questions [10]. First, discrimination between these pain phenotypes can be challenging for clinicians since patients can fit into more than one pain phenotype (e.g., mixed type) since one type (i.e., neuropathic) does not exclude another phenotype (i.e., nociplastic) [11]. Second, clinical identification of the presence of altered nociceptive pain processing for classifying a phenotype as phenotype is difficult since no gold standard exists for classifying heightened pain responses and no standardized tools have been defined. In 2021, the IASP proposed a set of clinical criteria and a grading system for classifying these three pain phenotypes [12]. These criteria are comprehensive, robust, properly developed, and with a proper potential to be applied in clinical practice [13]. Although CTS will be primarily considered a neuropathic condition, the IASP nociplastic criteria can be also present in some patients, leading to a mixed-type phenotype pain.

The identification of patients with a nociplastic pain phenotype has the potential to improve precision pain medicine practices in musculoskeletal pain conditions [14]. In this position paper, an international group of experts in CTS propose a clinical rationale for the application of the 2021 IASP clinical criteria to identify if a patient with CTS has a neuropathic and/or nociplastic pain phenotype. Proper distinction between pain phenotypes is important because the nociplastic type is more difficult to treat than other pain phenotypes. In fact, some interventions with a high probability of success in neuropathic pain could be less effective or even exacerbate symptomatology in patients with the nociplastic pain phenotype [14].

## 2. Phenotyping Carpal Tunnel Syndrome

### 2.1. Nociceptive Pain Phenotype

Nociceptive pain is defined as pain attributable to the activation of the peripheral receptive terminals of primary afferent neurons in response to noxious chemical, mechanical or thermal stimuli, and clinically, the pain response is proportional to the nociceptive input [15]. The fact that nerve pain is associated with the activation of peripheral nociceptors could support a nociceptive component in nerve-related pain. Nerve trunk pain is usually ascribed to an increased activity in sensitized nociceptors in the “nervi nervorum”, the nerve that innervates the connective tissue layers of the nerve itself [16]. In fact, there is evidence of the involvement of both nociceptive and non-nociceptive fibers contributing to the different symptoms experienced in CTS [17]. The lack of an association between clinical and neurophysiological signs of median nerve damage and sensory and motor symptomatology may be related to the involvement of nociceptive C-fibers in people with CTS [18] and the fact that the traditional clinical electrophysiological test assesses primarily the functioning of the thick non-nociceptive afferents. This hypothesis is supported by the fact that extra-median symptoms (nociplastic section) are not associated with electrical nerve conduction, suggesting a nociceptive component in CTS-related pain [19]. Nevertheless, the presence of median nerve damage clearly supports that CTS, by definition, is a neuropathic pain phenotype. The peripheral component of entrapment neuropathies, such as CTS, is strongly supported by the current literature [20].

An important topic to be considered is the presence of symptoms compatible with CTS but in the absence of electromyographic/electrophysiological changes. Qerama et al. identified that muscle-referred pain elicited by the infraspinatus muscle was able to mimic symptoms compatible with CTS but in the absence of nerve conduction studies [21]. In this sample, the muscle, but not the nerve, elicited sensory-related symptoms in the territory innervated by the median nerve, mimicking symptoms compatible with CTS. It is probable that these patients will exhibit a predominantly nociceptive phenotype since no current median nerve damage is still identifiable by gross electrophysiological assessments.

In individuals with a nociceptive pain phenotype, the relevance of the peripheral trigger is well established, and its treatment, if identifiable and responsive to management, would lead to clinical improvement. In Gifford’s mature organism model, exercise and manual therapies are claimed to be effective when the pain phenotype is nociceptive [22]. From a clinical viewpoint, in patients with the nociceptive phenotype, functional activity and early treatments targeting the peripheral input should be applied. However, current evidence supporting the use of physical therapy modalities applied just locally to the trigger area (i.e., the carpal tunnel), such as low-level laser therapy [23] or therapeutic ultrasound [24], does not support a potential benefit for these patients. These results reinforce that CTS is, by definition, a neuropathic pain phenotype.

### 2.2. Neuropathic Pain Phenotype

The Neuropathic Pain Special Interest Group (NeuPSIG) of the IASP defined pain as being of neuropathic origin when (1) there is lesion/disease of the somatosensory nervous system, either the peripheral or central nervous system; (2) symptoms are limited to a neuroanatomically plausible distribution of the nervous system; and (3) pain is supported by examination findings as well as laboratory and/or imaging [25].

Several clinical diagnostic tests are used for diagnosing CTS. A recent meta-analysis has pooled the following diagnostic odds ratio (dOR) for several test commonly used in clinical practice for the screening of CTS: Durkan test (dOR 15.84, 95%CI 3.78–66.38), Phalen test (dOR 7.23, 95%CI 4.06–12.86) and Tinel test (dOR 5.31, 95%CI 3.49–8.09) [26]. Additionally, the American Association of Electrodiagnostic Medicine, the American Academy of Neurology, and the American Academy of Physical Medicine and Rehabilitation Guideline proposes the following nerve conduction values for being considered positive: 1, median nerve sensory conduction velocity <40 m/s; and 2, median nerve distal motor latency > 4.20 [27]. Some authors have also proposed a classification on minimal, mild/moderate or severe CTS based on the presence of electrodiagnostic findings [28,29]. Accordingly, individuals with CTS will be always classified “a priori” as a neuropathic pain phenotype.

A positive diagnosis of CTS should be accompanied with negative electromyographic findings in the ulnar and radial nerves [27]; however, it is important to consider the presence of the median-to-ulnar nerve communicating branch in some patients with CTS [30]. Accordingly, electromyographic examination should be complementary to the presence of symptoms and physical examination.

### 2.3. Nociplastic Pain Phenotype

According to the IASP definition of nociplastic pain, sensitization of the central nervous system (e.g., increased responsiveness of nociceptive pain neurons within the central nervous system to their normal or subthreshold afferent input [31]) is thought to be the main underlying mechanism of this phenotype [9].

In the last decade, several studies have identified the presence of altered nociceptive pain processing in CTS. It has been identified that patients with CTS exhibit widespread pressure pain hypersensitivity [32], bilateral thermal pain hyperalgesia [33], bilateral and generalized elevated vibration thresholds [34], enhanced wind-up [35], impaired conditioned pain modulation [36] but not temporal summation [37]. In addition, several of these hyperalgesic responses are similar in those patients with minimal, moderate or severe CTS, suggesting that the damage of the median nerve seems to not be specific but to include additional mechanisms [38]. Nevertheless, a meta-analysis found significant generalized reduction in the mechanical, thermal or vibration detection thresholds (consistent results), generalized pain sensitivity to mechanical pain (heterogeneity results) and localized sensitivity to thermal pain (consistent results) in individuals with CTS [6]. Further, this review also showed that conditioned pain modulation is impaired in CTS [6]. It was concluded that hypoesthesia and increased thermal and mechanical pain are the dominant sensory phenotype in CTS but with inconclusive evidence about thermal and mechanical pain thresholds [6].

Additionally, patients with CTS, albeit those with minimal affection, also exhibit other disturbances associated with altered nociceptive processing, such as an impairment in left/right judgment of the affected extremity [39], adaptive changes in the homeostasis of memory T cells [40], or maladaptative brain changes [41,42]. These studies hypothesized that long-lasting sensory pain symptoms (peripheral drive) promote the blurring of median nerve-innervated digit representations through neural mechanisms (central drive) [39,41,42]. Similarly, other central nervous system-derived symptoms related to neuro-immune alteration, such as fatigue, poor sleep quality, or psychological disturbances, are also typical of nociplastic pain conditions [43] and have also been seen in individuals with CTS [44,45,46]. In fact, the presence of depressive or anxiety levels have been found to be indicators of poor post-surgery [47] or poor conservative [48] outcomes in CTS. Still, altered nociceptive processing and central sensitization are also considered underlying mechanisms of neuropathic pain.

Although current evidence would support the presence of altered nociceptive processing in patients with CTS, it is possible that subgroups exist and that not all patients exhibit altered nociceptive gain. In fact, it has been hypothesized that widespread sensory changes associated with sensitization are present in patients with CTS exhibiting extra-median symptoms, i.e., also neck pain, and not in those with just median nerve symptoms [49]. Interestingly, the presence of a comorbid neck condition or extra-median (multisite pain) symptomatology are associated with higher risk of requiring surgery [50]. Accordingly, the degree of nociplastic pain in patients with a neuropathic phenotype could be inversely related to clinical improvement from peripheral-based treatment. Additionally, it is possible that the presence of nociplastic pain could explain the presence of some post-surgical complications, such as complex regional pain syndrome [51].

## 3. Clinical Criteria/Grading System for Nociplastic Pain and Carpal Tunnel Syndrome

This section presents the clinical reasoning process applying the IASP criteria for identifying a predominant neuropathic or mixed-type nociplastic phenotype in patients with CTS [12]. It should be considered that one patient can fulfill the criteria for more than one pain phenotype, and also a patient with a neuropathic phenotype can exhibit some features of the nociplastic phenotype; therefore, it is needed to first determine whether the phenotype is predominant at a particular moment in a patient and follow with time.

### 3.1. Step 1—Duration of Pain

The first requirement, according to IASP clinical criteria, is that symptoms are present for at least 3 months. The natural history of CTS is chronic and highly fluctuating with patients exhibiting symptoms over years [52]; accordingly, this criterion is fulfilled in most patients.

### 3.2. Step 2—Distribution of Pain

**Figure 1:**
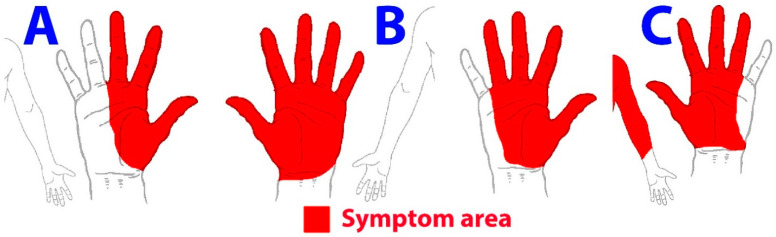
Pain symptoms within the median nerve-innervated territory (**A**), globe distribution (**B**) or extra-median nerve symptoms (**C**).

A nociceptive pain pattern is usually discrete and localized, with neuroanatomical sense, and is generally exacerbated with defined pain triggers (specific movements and activities). The most common symptoms experienced by individuals with CTS include pain and/or paresthesia in areas innervated by the median nerve, i.e., the thumb, index, and/or middle fingers (Figure 1A). Symptoms worsen during activities involving the hand/wrist but also at night. Since patients with CTS exhibit evidence of a lesion of the median nerve, the predominant phenotype is neuropathic as previously discussed. Interestingly, paresthesia, but not pain, is the symptom most commonly associated with neurophysiological damage of the median nerve [53].

A nociplastic pain pattern is more generalized or widespread [12]. It is commonly seen in clinical practice that patients with CTS exhibit symptoms not only in those areas innervated by the median nerve but also in extra-median nerve areas. Zanette et al. observed that 35% of patients with CTS exhibit a glove distribution of their symptoms (Figure 1B) [54], and that 45% of the patients also report pain in proximal areas of the upper extremity, including the elbow or the shoulder (Figure 1C) [55]. A study, using an electronic software for analyzing the pain extent, found that 88% of women with CTS reported extra-median pain symptoms [56]. Nevertheless, some studies observed that extra-median symptomatology is not associated with more nerve damage since individuals with severe CTS report more median nerve symptoms, whereas the presence of extra-median symptoms is more frequent in those with minimal or moderate CTS [19,57]. Accordingly, a careful clinical assessment and interpretation of the patient’s pain pattern during the treatment process is needed to identify the evolution of the symptoms from a localized to a more generalized pattern.

### 3.3. Step 3—Determine Whether Nociceptive Pain Is Present

The next step is to identify if the pain can be entirely explained by nociceptive mechanisms [12]. In such a way, imaging techniques should identify a source of nociception (different from nerve) that potentially can be (partly) responsible for the patient’s pain pattern. For instance, proper ultrasound examination could reveal the presence of possible tenosynovitis of the flexor tendons at the wrist level, which can mimic symptoms compatible with a potential nerve entrapment [58]. Another potential nociceptive-related pain would be the presence of a trigger finger, a condition which can be also properly diagnosed with ultrasound imaging [59]. It is unlikely that a patient with a diagnosis of CTS would exhibit a source of nociceptive different than the median nerve. In fact, a nociceptive pain phenotype could be only considered in people with CTS-related symptoms, but with negative electromyographic findings (e.g., referred pain from shoulder muscles as reported by Qerama et al. [21]) or in those with localized tendon damage at the wrist level [58,59]. It is important to note that identification of a source of nociception does not exclude the possibility of concomitant neuropathic or nociplastic pain (mixed-type pain).

### 3.4. Step 4—Determine Whether Neuropathic Pain Is Present

The fourth step is to identify if symptoms cannot entirely be explained by the neuropathic pain mechanism [12]. As previously commented, people with CTS present clear evidence of altered nerve conduction of the median nerve (e.g., lesion on somatosensory nervous system). This situation would lead to a neuropathic phenotype, the common classification of CTS. Indeed, sustained neuropathic pain also results in increased hyperexcitability of the central nervous system [60]. The progression from a neuropathic phenotype into a nociplastic (or mixed type) pain phenotype provides one explanation for the spreading symptomatology beyond the innervation territory of the median nerve. This evolution is based on the fact that nerve-related pain represents a driver in the development of altered nociceptive processing and, hence, long-lasting nociception from the median nerve could facilitate central sensitization changes.

### 3.5. Step 5—Elucidate the Presence of Pain Hypersensitivity

The fifth step involves screening for signs of pain hypersensitivity, including hyperalgesic (defined as an exaggerated pain response to painful stimuli) and allodynic (defined as pain in response to stimuli that normally do not elicit pain) responses [12]. As it was previously commented, evidence has shown the presence of generalized mechanical, thermal and vibration hypoesthesia, generalized mechanical hyperalgesia and localized thermal pain hyperalgesia in individuals with CTS [6]. However, the heterogeneity in results also supports the presence of potential subgroups of patients. Similarly, the presence of altered conditioned pain modulation [36] also supports the presence of sensitization. According to the IASP clinical criteria, if the first five steps are positive for nociplastic pain, a patient could be classified with “possible nociplastic pain” [12].

### 3.6. Step 6—Check for the History of Pain Hypersensitivity

Step 6 proposed that if a patient reports symptoms of pain hypersensitivity, particularly allodynic responses, with daily living activities, it can be considered “probably nociplastic pain” [12]. Allodynic responses are expected in patients with CTS and, in fact, they are commonly reported in clinical practice, but no study has investigated this.

### 3.7. Step 7—Determine If Comorbidities Are Present

The final step involves screening for sensitivity to other stimuli, including sensitivity to other stimuli, such as sound, light, or odors, and the presence of underlying comorbid medical conditions or other central nervous system-associated symptoms, e.g., poor sleep quality, fatigue and cognitive problems [12]. For instance, different underlying medical conditions, such as diabetes [61], rheumatoid arthritis [62] or hereditary transthyretin amyloidosis with polyneuropathy [63], are considered to be potential risk factors of CTS. In addition, as it was previously commented, the presence of psychological disturbances, e.g., depression, and poor sleep is present in almost 40% of subjects with CTS [44,45,46]. The presence of these conditions could be related to specific medication consumption, which could also alter pain sensitivity; accordingly, a history of medication consumption is also needed. If this criterion is also fulfilled, CTS-related pain should be classified as “probable nociplastic pain” [12]. Finally, another clinical situation is that CTS can also be co-morbid with a nociplastic pain condition, e.g., fibromyalgia. In fact, different studies have associated the presence of CTS in individuals with fibromyalgia syndrome [64,65].

**Figure 2:**
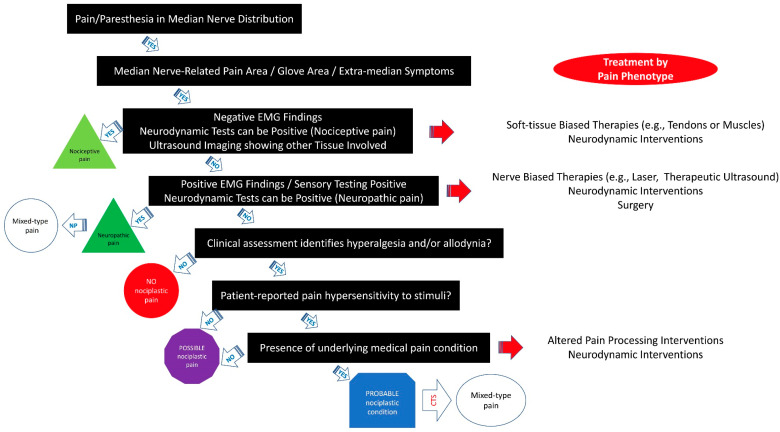
Clinical decision-making tree of the IASP clinical criteria for patients with carpal tunnel syndrome (CTS).

Figure 2 provides a clinical decision-making tree for clinicians based on the IASP clinical criteria for assessing the pain phenotype in individuals with CTS-related symptoms. It is important to note that the reliability and validity of the 2021 IASP clinical grading criteria [12] have not been evaluated. Additionally, more research is needed to determine the prognostic value and responsiveness of the IASP clinical criteria on treatment outcomes in clinical trials in individuals with CTS.

## 4. Considering Pain Phenotype in Carpal Tunnel Syndrome Treatment

No current consensus exists on which therapeutic option should be applied as the first-line treatment for managing individuals with CTS. Surgery and conservative approaches are recommended in clinical practice guidelines [66]. The challenge facing clinicians is how to determine the proper treatment approach for each individual patient with CTS-related pain, as patients are likely to be different in their treatment response depending on the potential pain phenotype.

Most treatments proposed for managing CTS are mainly targeted on the carpal tunnel. In fact, surgery continues to be the most common treatment approach proposed for individuals with CTS [67]. Both open and endoscopic surgical interventions provide similar clinical results [68], and although surgery provides positive long-term results, the recurrence rate is estimated to be around 30% [69].

Similarly, several conservative interventions targeting the carpal tunnel area have been proposed for managing CTS: splints [70], corticoid injections [71], nerve gliding exercises [72], therapeutic ultrasound [24], transcutaneous electrical nerve stimulation (TENS) [73], low-level laser [23], or local manual therapy [74]. Shi et al. concluded that differences between conservative and surgical treatments are smaller than expected [75]. Discrepancies between the published studies can be explained by the fact that most of them apply interventions just focused on the carpal tunnel (peripheral-drive approach), considering CTS as a neuropathic pain phenotype, but without considering the possibility of a nociplastic or mixed-typed phenotype. In fact, centrally mediated symptoms are associated with worse functional outcomes after surgery [76]. Therefore, the application of IASP criteria [12] in individuals with CTS could allow clinicians to improve treatment strategies according to their pain phenotype. In such a scenario, when developing a treatment plan, the identification of the presence of a nociplastic pain phenotype should be included into the clinical decision tree (Figure 2).

Current evidence supports that the peripheral drive due to median nerve damage may initiate, activate, and maintain central sensitization processes. In such a scenario, a neuropathic pain phenotype can evolve to the nociplastic pain phenotype. This could be particularly present in those patients developing extra-median symptoms as previously suggested [54,55,56]. This hypothesis agrees with findings from previous studies observing that extra-median symptoms are not associated with electrodiagnostic findings and not present in patients with severe CTS [19,57]. Current reasoning would support clinical findings that conservative treatments could be mostly applied as the first-line option in individuals with less nerve involvement (more centrally mediated/more nociplastic phenotype), whereas surgery would be the first line in those with worse nerve affectation (more peripherally mediated/pure neuropathic phenotype).

Accordingly, the clinical management of patients with CTS needs to extend beyond the local tissue pathology (i.e., median nerve entrapment) and to incorporate strategies directed at normalizing altered nociceptive pain processing (if needed) since removing the peripheral drive from the median nerve at the carpal tunnel potentially might be able to modulate the nervous system but only partially. Therefore, treatment of the neuropathic pain phenotype will include tissue-based interventions, e.g., bottom–up techniques, whereas management of the nociplastic pain phenotype should also include nervous system interventions, e.g., top–down techniques [77]. Figure 2 includes potential interventions according to the predominant pain phenotype. This clinical reasoning was applied in a randomized clinical trial investigating the effect of manual therapies, including desensitization maneuvers of the central nervous system against surgery in a sample of women with CTS [78,79]. The results from this clinical trial showed that the application of manual therapies targeting not only the carpal tunnel area but also all of the upper extremity, and following the proposed clinical reasoning in this paper, exhibited better short-term (at one and three months) and similar long-term (one and four years after) effects on pain and function outcomes compared to surgery [78,79]. Nevertheless, this clinical trial did not identify the predominant pain phenotype in these patients. Future clinical trials determining the treatment by applying the IASP criteria for identifying the predominant pain phenotype in individuals with CTS are now needed.

## 5. Conclusions

Carpal tunnel syndrome is traditionally classified as a neuropathic condition with or without pain. The current paper summarizes data supporting the possibility of subgrouping individuals with CTS-related pain into nociceptive, neuropathic, nociplastic or mixed-type phenotypes. It is concluded that CTS is a neuropathic condition but can also be comorbid with a nociplastic pain condition. The presence of spreading pain (extra-median) symptoms and the development of an altered pain processing seem to be potential signs of nociplastic pain. In the latter cases, the development of a nociplastic phenotype would explain the lack of clinical effect of treatment interventions targeting just the carpal tunnel area. We propose a clinical decision tree by using the 2021 IASP classification criteria for identifying the predominant pain phenotype in individuals with CTS-related pain, albeit CTS being a priori a neuropathic pain condition. The identification of nociplastic-associated pain in patients with CTS requires a more nuanced multimodal treatment approach to achieve better treatment outcomes.

## Data Availability

Not applicable.
